# Integrated bioinformatics and molecular docking identify novel key genes, pathways, and potential candidate drugs in papillary thyroid carcinoma

**DOI:** 10.1016/j.jgeb.2026.100791

**Published:** 2026-08-08

**Authors:** Mehdi Koushki, Masoumeh Farahani, Nasrin Amiri-Dashatan, Fatemeh Fateminasab, Reza Fadaei, Hossein Chiti, Somayeh Chahkandi, Lida Perseh

**Affiliations:** a Cancer Gene Therapy Research Center, Zanjan University of Medical Sciences, Zanjan, Iran; b Department of Clinical Biochemistry, School of Medicine, Zanjan University of Medical Sciences, Zanjan, Iran; c Proteomics Research Center, Shahid Beheshti University of Medical Sciences, Tehran, Iran; d Zanjan Metabolic Diseases Research Center, Health and Metabolic Diseases Research Institute, Zanjan University of Medical Sciences, Zanjan, Iran; e Department of Physical Chemistry, Faculty of Chemistry, University of Mazandaran, Babolsar, Iran; f Department of Pharmacology, Vanderbilt University, Nashville, TN, USA

**Keywords:** Papillary thyroid carcinoma (PTC), Gene expression, Biomarker, Drug discovery, Molecular docking

## Abstract

Papillary thyroid carcinoma (PTC) is the most prevalent form of well-differentiated cancer of the thyroid gland. In the current study, we applied integrated bioinformatics analyses to introduce key genes with diagnostic and prognostic value, and decipher the signaling pathways involved in patients with PTC. We analyzed four integrated profiles from Gene Expression Omnibus (GEO) including, GSE58545, GSE3467, GSE29265, and GSE60542. GEO2R was utilized to analyze and detect differentially expressed genes (DEGs), and then the results of four datasets were integrated. Gene ontology (GO), KEGG-related pathway, protein-protein interaction (PPI) network, survival, and immunohistochemical assessment were performed by DAVID, ShinyGO, STRING, GEPIA, and Human Protein Atlas (HPA), respectively. In addition, we performed molecular docking of hub genes with 1615 FDA approved drugs. The findings of GO enrichment indicated that the up- and down-regulated genes were mostly participating in cell adhesion, extracellular region, and bicarbonate transport, proteinaceous extracellular receptor complex, respectively. Pathway enrichment indicated that the upregulated genes were linked with ECM-receptor interaction, and downregulated genes were largely involved in tyrosine metabolism and the JAK/STAT signaling pathway. The FN1, DPP, CD36, KIT, and ITGA2 proteins and hsa-mir-124-3p were identified as druggable target genes. According to docking analysis, Olysio, Gabapentin, Naldemedine, Imatinib and Enzacamene were drugs that exhibited strong interactions with the target proteins. The findings of the current study offer that FN1, DPP4, CD36, KIT, ITGA2 and hsa-miR-124-3p may be novel biosignatures and therapeutic targets for PTC. In addition, it was demonstrated that discovered pharmaceuticals may be employed as prospective PTC treatments.

## Highlights

• Papillary thyroid carcinoma (PTC) is the most prevalent form of well-differentiated cancer of the thyroid gland.
• Integrated in silico analyses were employed to identify the key genes and signaling pathways involved in PTC.
• GO analysis showed that dysregulated genes were involving in cell adhesion, extracellular region, and bicarbonate transport.
• Docking revealed strong interactions between target proteins and Olysio, Gabapentin, Naldemedine, Imatinib, and Enzacamene.
• The key genes of FN1, DPP4, CD36, KIT, ITGA2 and hsa-miR-124-3p may serve as new biomarkers and therapeutic targets for PTC.

## Graphical abstract

**Figure:**
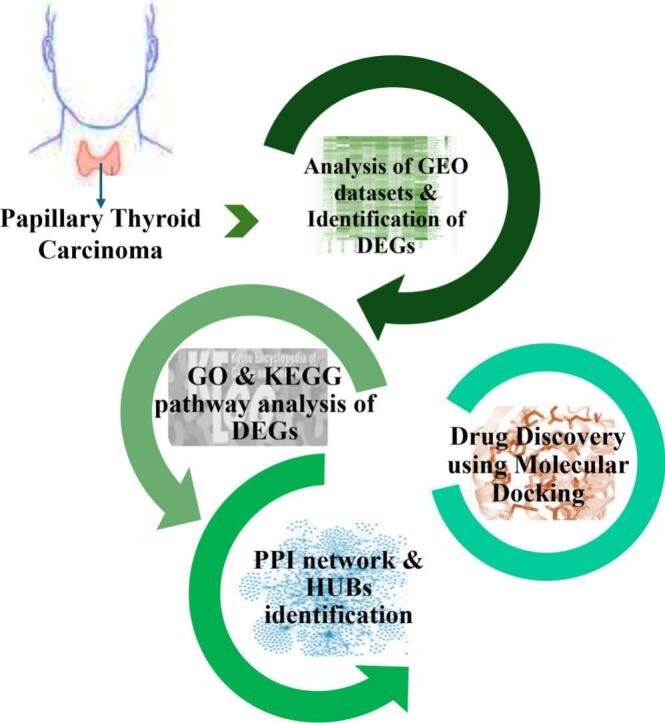

## 1 Introduction

The thyroid is a major endocrine gland that uses iodine to release hormones that control various important tasks including heart rate, blood pressure, body heat, and especially the basic level of metabolic rate. Thyroid cancer (TC) stands as the primary malignancy of the endocrine system and currently ranks as the 13th most commonly diagnosed cancer worldwide, in 2022 in the U.S., and the 6th most commonly detected cancer among women.^1^ Additionally, TC Thyroid cancer is categorized into four main types that about 70 to 80% of thyroid cancers are papillary thyroid carcinomas (PTCs), the most prevalent kind of malignant thyroid cancer.^2^PTC is an epithelial malignancy along with differentiation of follicular cells and a set of distinct histopathological characteristics. The overall prognosis of PTC is good, especially for those under 45 years old.^3^ The methods of thyroid cancer treatment are surgery, treatment with radioactive iodine (^131^I), treatments based on molecular targets tyrosine kinase inhibitors (TKIs), and standard treatments vary according to the type and stage of the tumor.^2^ Over the recent years, bioinformatics analyses have emerged as essential tools in life science, which can be utilized for early diagnosis, improved prognosis, and clinical management of human diseases, including cancer.^4^, ^5^, ^6^, ^7^ Furthermore, developments in microarray techniques have led to the creation of an unparalleled amount of expression data. The microarray profiling method is a large scale approach for identifying gene expression in tissues at the mRNA level, which has significantly emerged as a valuable method in the field of medical oncology.^8^, ^9^ Several studies have used this approach to identify DEGs between cancerous tissue samples and adjacent normal tissue in papillary thyroid carcinoma cases. For instance, Kim et al. studied genetic profiles in the Korean population with PTC by cDNA microarray method and presented TFCP2L1, LYVE-1, and KLK7 genes that were not previously identified in the PTC.^10^ In another study, Gubala et al. identified that dipeptidyl peptidase 4 (DPP4), tissue inhibitor of metalloproteinase 1 (TIMP1), and fibronectin 1 (FN1), is a novel molecular panel for PTC.^11^ By investigating the dysregulated gene expression profiles in tumor versus control tissues, it is possible to elaborate the molecular mechanisms of diverse malignancies, such as PTC. Such insights enable the discovery of novel therapeutic targets and key signaling pathways for precision medicine. In this field, there is a limited comprehensive analysis based on GEO datasets for PTC. Despite the progress in understanding PTC, a comprehensive integration of multi-platform transcriptomic data to identify synergistic therapeutic targets remains limited. Our research is based on the premise that the oncogenic development of PTC is influenced by a coordinated disruption of a particular network of hub genes. We propose that addressing these interconnected nodes with multi-target pharmacological agents, as opposed to conventional single-target inhibition, may provide a more effective therapeutic strategy. To evaluate this premise, we set out to identify crucial hub genes through bioinformatic analysis of GEO datasets and subsequently employed molecular docking techniques to identify the most promising multi-target candidate drugs. This comprehensive approach aims to elucidate the molecular mechanisms underlying PTC and to present new molecular targets and effective drug alternatives for enhanced diagnosis, prognosis, and treatment.

## 2 Materials and methods

### 2.1 Dataset selection

We conducted a search in the GEO (https://www.ncbi.nlm.nih.gov/geo/). The following words were searched in this database: “thyroid cancer”, “thyroid carcinoma”, and “papillary thyroid carcinoma”. After the screening, four datasets including GSE58545, GSE3467, GSE29265, and GSE60542 were selected. These datasets were selected as validation datasets because they possess an adequate sample size, well-balanced disease and controls, and consistent tissue sources that align with the discovery datasets. GSE58545 dataset was used GPL96 [HG-U133A] Affymetrix Human Genome U133A Array, and the others (including GSE3467, GSE29265, and GSE60542) were according to GPL570 [HG-U133_Plus_2] Affymetrix Human Genome U133 plus 2.0 Array. The complete information about the selected papillary thyroid cancer datasets in GEO including, normal and cancerous sample sizes is shown in Table 1.

**Table 1:** The detailed information of four studied datasets.

Datasets	Platform	Sample	Normal (n)	PTC (n)	Up-regulated genes (n)	Down-regulated genes (n)	Total DEGs (n)	Country	First Author (year)
GSE58545	GPL96 [HG-U133A] Affymetrix	thyroid	18	27	246	249	495	Poland	Rusinek D et al., (2015)
GSE3467	GPL570 [HG-U133_Plus_2] Affymetrix	thyroid	9	9	180	139	319	USA	He et al., (2005)
GSE29265	GPL570 [HG-U133_Plus_2] Affymetrix	thyroid	20	20	196	183	379	Belgium	Tomas et al., (20111)
GSE60542	GPL570 [HG-U133_Plus_2] Affymetrix	thyroid	30	33	253	245	498	Belgium	Tarabichi et al., (2015)
Total	–	–	77	89	875	816	1691	–	–

#### 2.1.1 Inclusion and exclusion criteria

To ensure the biological consistency and statistical robustness of the meta-analysis, a set of stringent inclusion and exclusion criteria was applied to the datasets retrieved from the Gene Expression Omnibus (GEO) database. The inclusion criteria were defined as follows: (i) Disease specificity: only datasets specifically focusing on Papillary Thyroid Carcinoma (PTC) were included to minimize biological noise. (ii) Technological compatibility: datasets were selected based on the use of compatible high-throughput platforms (e.g., microarray or RNA-Seq) to facilitate standardized normalization and seamless integration. (iii) Sample size and quality: only datasets with a sufficient number of biological replicates and high-quality expression profiles were considered, ensuring adequate statistical power for differential expression analysis. (iv) Clinical metadata availability: To enable subsequent survival analysis and clinical correlation, datasets must have provided relevant clinical information (e.g., disease stage or patient survival status). Datasets that did not meet all the aforementioned criteria, or those involving heterogeneous thyroid cancer subtypes that could confound the results, were excluded from the study.

### 2.2 Identification of genes with differential expression

The analysis of every dataset based on differential expression was conducted using the GEO2R analysis tool between PTC and regular thyroid tissues. The datasets were normalized and Benjamin–Hochberg (FDR) correction was performed to adjust the *p*-values. Genes with expression variations between types of tissue with adjusted p-value <0.05 and log fold-change (LogFC) ≥ 1.5 were defined as significant DEGs. DEGs were defined based on this threshold to control the False Discovery Rate (FDR) and minimize false positives, ensuring that the selected genes exhibited both biological relevance and statistical significance. Statistical analyses were conducted on datasets separately. After detection of differentially expressed genes (DEGs) in each dataset, the common genes that are either up-regulated or down-regulated across four datasets were analyzed by the Venny platform to identify the overlapping regions among the analyzed datasets.

### 2.3 GO enrichment and KEGG pathway analysis for DEGs

To decipher the molecular function (MF), biological process (BP), and cellular component (CC) related pathways of the DEGs in papillary thyroid carcinoma, GO was done using the DAVID application (https://davidbioinformatics.nih.gov/), and KEGG pathway analyses in ShinyGO 0.80 (http://bioinformatics.sdstate.edu/go80/). GO and KEGG analysis results were screened according to *p*-value <0.05.

### 2.4 PPI network building and hubs screening

The PPI network was built using all the integrated upregulated and downregulated genes by STRING web-based tool (https://string-db.org). The Cytoscape version 3.7.0 was utilized for analysis of the constructed network. Then, through the CytoHubba plugin, we chose the top 20 genes with the highest connectivity rank or node degree in the network as the key (hub) genes.

### 2.5 Survival analysis for hub genes

To further elucidate the association between hubs and PTC prognosis, we performed survival analysis using Gene Expression Profiles Interactive Analysis (GEPIA, http://gepia.cancer-pku.cn). The *p*-value <0.05 was determined statistically significant due to its importance for PTC prognosis, and then these central genes were introduced as key genes.

### 2.6 Confirmation of hubs in the TCGA database

GEPIA delivers quick functional information. In this work, to verify key genes, we utilized GEPIA and evaluated their expression in TCGA. For the DFS (Disease-Free Survival) analysis, we utilized the THCA dataset from TCGA via the GEPIA platform. The expression levels were dichotomized into ‘high’ and ‘low’ groups using the median cutoff value. The Cox Regression model was used to analyze a gene based on gene expression. We reported the results in the form of a boxplot. The criteria of our screening were LogFC ≥1.5 and *p*-value <0.05.

### 2.7 miRNA-key genes network creation

miRNet (https://www.mirnet.ca/) was applied to discover the associated miRNAs with selected key genes (including FNA1, DPP4, CD36, KIT, and ITGA2). In addition, Cytoscape was applied to envisage the miRNA–key gene network.

### 2.8 Immunohistochemical (IHC) analysis

Because of the high affinity between antibodies (Ab) and antigens (Ag), we unified a method based on antibody with gene expression data to provide a comprehensive expression profile. Expressed protein levels of selected key genes in normal and PTC samples were measured according to the Human Protein Atlas database (HPA, https://www.proteinatlas.org).

### 2.9 The process of virtual screening alongside docking

To discover potential new drugs for the PTC therapy, we carried out a virtual screening and docking procedure on the all drugs with FDA approval and key genes (proteins) by the AutoDock Vina package in the PyRx program Version 0.8.^12^ The 3D (three-dimensional) structure of key (hub) genes was extracted from the Protein Data Bank (PDB: https://www.rcsb.org/) as an online resource.^13^ Additionally, we retrieved the 3D structures of drugs, totaling 1615 ligands, from the ZINC at http://zinc.docking.org/ in the format of SDF (retrieved on 3.30.2021).^14^

### 2.10 Analyzing and visualizing the outcomes of docking

Originally, the outcomes of docking were assessed by the PyRx program to evaluate binding affinity and binding energy between drugs (ligands) and proteins. The initial step involved removing water molecules from the .pdb and .sdf files, and adding any missing hydrogen atoms. PDBQT files were then created to prepare the coordinate information for the selected key proteins and for the drug structures. Subsequently, molecular docking was conducted between each of the genes as the receptors for each drug compound.

For further investigation of binding modes between proteins (acceptor) and drug compounds, rigid (receptor): flexible (ligand) docking was performed. To ensure biological relevance and computational accuracy, targeted docking was preferred over blind docking to focus the search space on biologically active regions, thereby reducing false-positive hits and improving the accuracy of binding affinity predictions. Five drugs with the best and highest interaction energy for each protein were selected and analyzed using the AutoDock 4.2 program. This analysis aimed to identify residues involved in interactions, employing an empirical free energy function and the Lamarckian Genetic Algorithm (LGA). AutoGrid was employed to produce the grids after assigning Gastieger charges to the macromolecule input files.^15^, ^16^ Binding energy was computed using ranking mechanisms, and the arrangements of every cluster were set from the lowest to the highest according to their binding energy. The docking pose with the lowest free energy of binding, which was the most populated, was selected as optimal. To validate the molecular docking procedure, a positive control was achieved using redocking the co-crystallized ligands with their respective binding sites, and the RMSD between the docked and crystallographic poses was evaluated. For proteins without co-crystallized ligands and apo forms (FN1 and ITGA2), no positive control was valid. Furthermore, a negative control was performed by human serum albumin (HSA, PDB ID: 1AO6) as an unrelated target through blind docking to compute interaction specificity.^17^ To more thoroughly examine the receptor-ligand binding mechanisms and create visual representations, BIOVIA Discovery Studio Visualizer 2021 was employed.

## 3 Results

### 3.1 Detection of DEGs

**Fig. 1:**
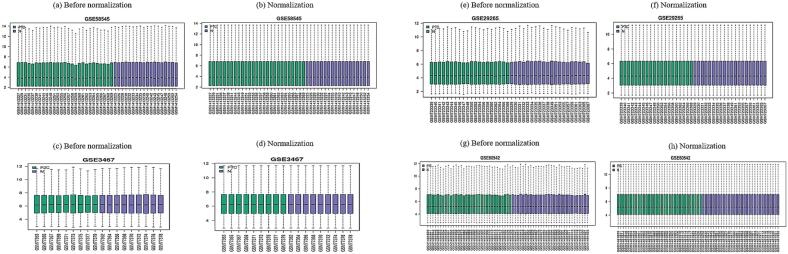
Data normalization and distribution analysis across different GEO datasets. To ensure comparability between datasets, expression profiles were normalized prior to differential expression analysis. (a–b) GSE58545; (c–d) GSE3467; (e–f) GSE29265; (g–h) GSE60542.

**Fig. 2:**
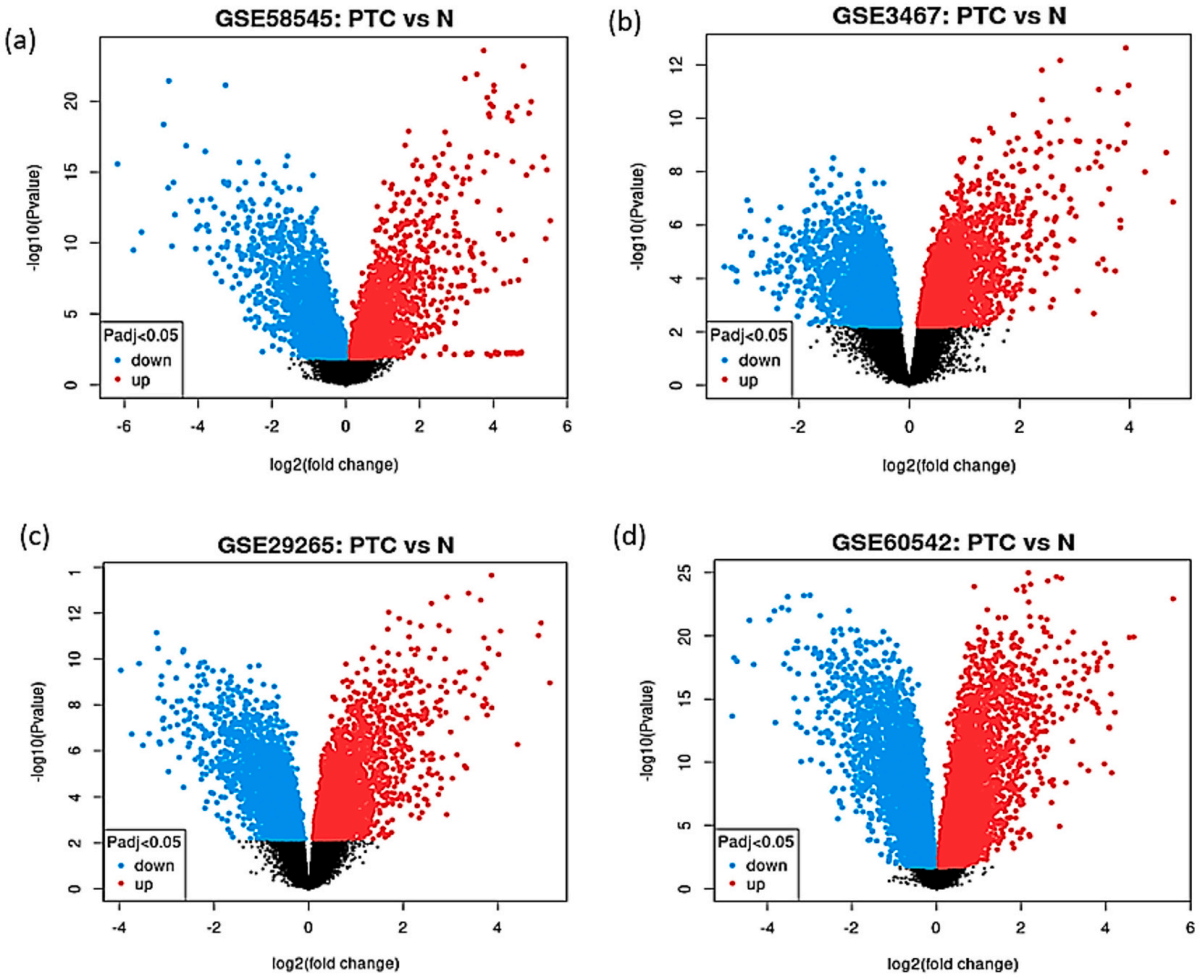
Volcano plots illustrating the differentially expressed genes (DEGs) across four independent GEO datasets. The plots demonstrate the distribution of gene expression changes based on statistical significance and magnitude of change. (a) GSE58545, (b) GSE3467, (c) GSE29265, (d) GSE60542. Red dots: up-regulated genes. Blue dots: downregulated genes. Black dots: genes with no significant differences in the expression level. (For interpretation of the references to colour in this figure legend, the reader is referred to the web version of this article.)

GSE58545 consists of 27 PTC and 18 normal samples, GSE3467 includes 9 PTC and 9 normal samples, GSE29265 contains 20 PTC samples and 20 normal samples, and GSE60542 comprises 33 PTC and 30 normal samples (Table 1). The thyroid carcinoma expression 4 datasets were normalized and analyzed. The before and after normalization graphs, and volcano plots of every dataset are presented in Fig. 1 and Fig. 2, respectively. 246 upregulated and 249 downregulated genes were recognized in GSE58545, while 180 and 139 up- and downregulated genes were identified in GSE3467, respectively. In GSE29265 dataset, 196 upregulated and 183 downregulated DEGs were detected, and 253 upregulated and 245 downregulated DEGs were selected in GSE60542 (Table 1).

### 3.2 Selection of common DEGs

**Fig. 3:**
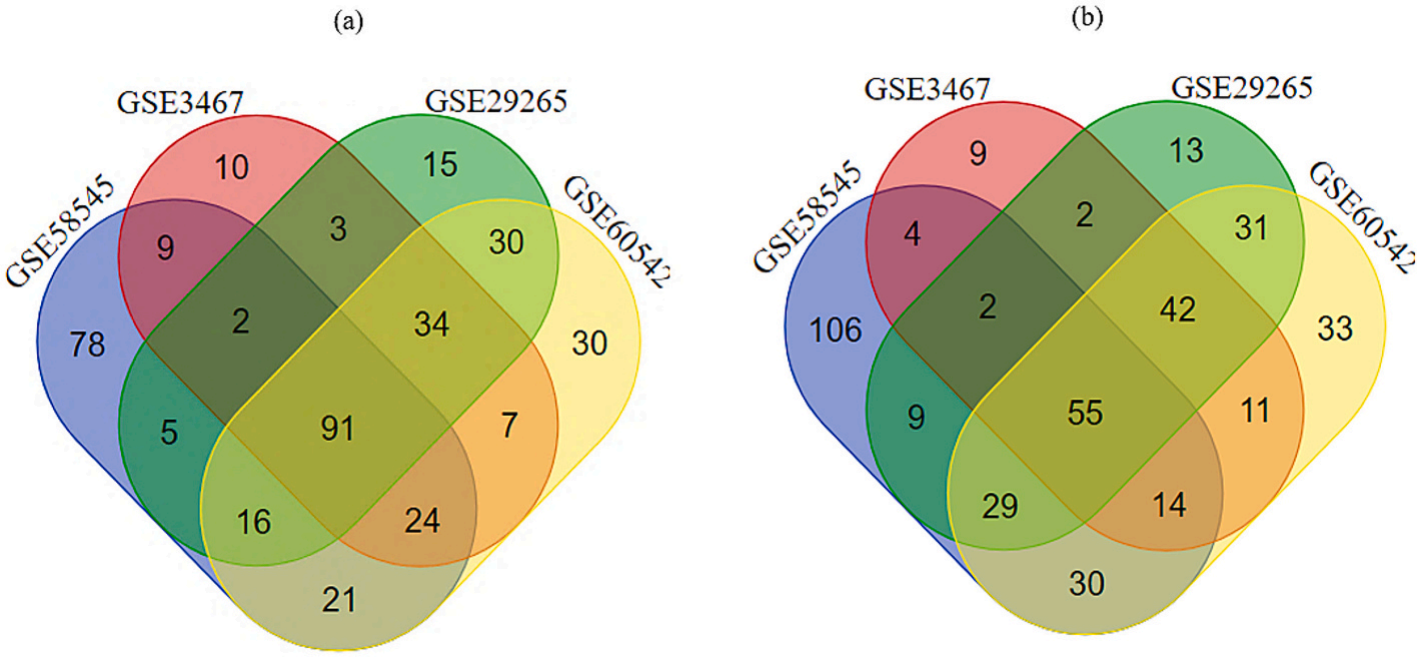
Identification of overlapping differentially expressed genes (DEGs) across four GEO datasets. The Venn diagram illustrates the intersection of DEGs identified from GSE58545, GSE3467, GSE29265, and GSE60542: (a) up-regulated genes; (b) down-regulated genes. A total of 146 common DEGs (91 up-regulated and 55 down-regulated) were identified for subsequent pathway analysis.

A total of 146 common DEGs (including 91 up- and 55 downregulated genes) were detected through Venn diagram analysis (Fig. 3). The list of total integrated differential expressed genes including up- and downregulated genes is presented in Table 2.

**Table 2:** Integrated DEGs in GEO datasets.

DEGs (logFC ≥ 1.5)	Gene names
Up-regulated (*n* = 91)	CDH3, KCNJ2, KRT19, LAMB3, PDZK1IP1, TENM1, BID, DIRAS3, RXRG, CXCL14, PLAU, MPZL2, ALDH1A3, GDF15, SLC27A6, ENTPD1, GALE, MET, PTPRE, KLK10, ALOX15B, SDC4, ALOX5, HEY2, ETV5, ABCC3, ST6GALNAC5, DUSP6, NRCAM, RUNX1, TMPRSS4, SLC34A2, SPP1, CD55, NMU, RAB27A, CFI, SLPI, LRP4, CLDN1, DPP4, GABBR2, DUSP4, APOE, CLDN10, PDLIM4, TYMS, GALNT7, YME1L1, SFN, IGSF1, COL8A2, DUSP5, TUSC3, PROS1, CST6, AHNAK2, TACSTD2, ITGA2, CAMK2N1, QPCT, SCEL, COMP, TESC, TIMP1, NELL2, P4HA2, PCSK2, PRSS23, SCG5, CHI3L1, AMIGO2, CITED1, SFTPB, SERPINA1, COL13A1, APOC1, TGFA, PLXNC1, ZMAT3, SLIT1, CYP1B1, DCSTAMP, HMGA2, TIAM1, FN1, PSD3, AGR2, DTX4, LAMP5, CTSC
Down-regulated (*n* = 55)	HBA2///HBA1, PKNOX2, CCL21, PPARGC1A, DEPTOR, MPPED2, DPT, CRABP1, ZFPM2, CITED2, SHANK2, ADH1B, ANK2, DIO1, TCEAL2, KIT, FABP4, RGS16, HGD, PAPSS2, GLT8D2, CA4, LRP1B, DGKI, CDH16, GHR, SLC26A4, LIFR, ZMAT4, SLITRK5, CSGALNACT1, LYVE1, MMRN1, AGTR1, TFCP2L1, CLMN, TNFRSF11B, CD36, IPCEF1, CWH43, MATN2, ELMO1, NCAM1, TPO, SLC4A4, TFF3, AOX1, GPM6A, BEX1, MT1M, PROM1, DPP6, HBB, FHL1, SORBS2

### 3.3 GO and KEGG pathway analyses for DEGs

**Fig. 4:**
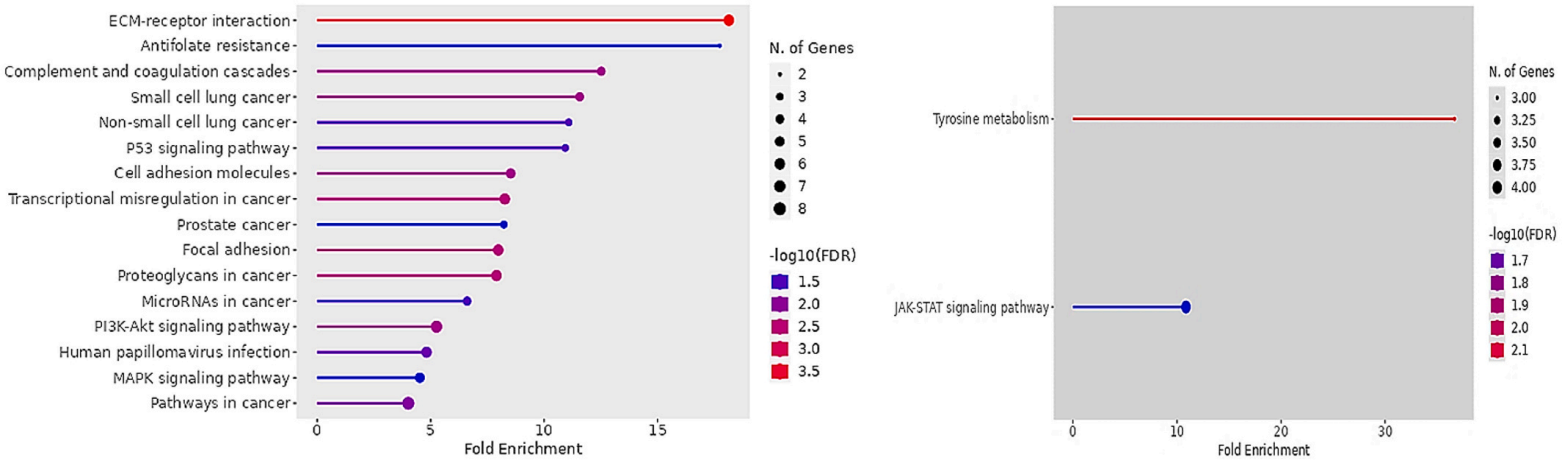
Functional enrichment analysis of integrated DEGs via ShinyGO. Identification of significantly enriched KEGG pathways associated with differentially expressed genes. **(a)** Top enriched KEGG pathways for up-regulated genes. **(b)** Top enriched KEGG pathways for down-regulated genes.

GO analyses of the DEGs were carried out by the DAVID tool, and the outcomes are summarized in Table 3. GO analysis indicated that in BP, the upregulated genes were firstly enriched for the GO terms including “Cell adhesion”, “Blood coagulation”, “Proteolysis”, “Cell-cell adhesion”, and “Peptide hormone processing”. CC analysis indicated that the DEGs were remarkably enriched in the “Extracellular region”, “Extracellular exosome”, “Extracellular space”, “Endoplasmic reticulum-Golgi intermediate compartment membrane”, and “Endoplasmic reticulum lumen”. Regarding MF, the up-regulated genes were enriched for “Serin-type endopeptidase activity”, “Heparan sulfate proteoglycan binding”, “Heparin-binding”, “Extracellular matrix structural constituent”, “Protein tyrosine/threonine phosphatase activity”. GO analysis of downregulated genes showed that genes enriched in BP terms including “Bicarbonate transport”, “Cell adhesion”, “Cellular oxidant detoxification”, “Receptor-mediated endocytosis”, and “Blood coagulation”. For CC the downregulated genes were enriched for the GO terms: “Receptor complex”, “Plasma membrane”, “Apical plasma membrane”, “Brush border membrane”, and “Membrane”. For MF the downregulated genes were enriched for the GO terms: “Fatty acid binding”, “Peroxidase activity”, “Growth factor binding”, “Cytokine binding”, and “RNA polymerase II Sequence-specific DNA binding TF binding”. As shown in Fig. 4 and Table 4, the significantly enriched KEGG pathways for upregulated genes were associated with ECM-receptor interaction and antifolate resistance (Fig. 4a). In addition, tyrosine metabolism and the JAK/STAT signaling pathway were significant signaling pathways associated with downregulated genes (Fig. 4b).

**Table 3:** GO analysis of integrated DEGs (logFC≥1.5).

Category	Term	Count	*p*-value	Fold enrichment	FDR
*Up-regulated genes top 5 enriched GO terms*					
GOTERM_BP_DIRECT	Cell adhesion	15	2.0E-7	5.9	2.0E-4
GOTERM_BP_DIRECT	Blood coagulation	6	6.1E-5	14.3	3.0E-2
GOTERM_BP_DIRECT	Proteolysis	9	1.0E-3	4.3	3.4E-1
GOTERM_BP_DIRECT	Cell-cell adhesion	6	1.8E-3	6.8	4.6E-1
GOTERM_BP_DIRECT	Peptide hormone processing	3	2.6E-3	39.0	5.1E-1
GOTERM_CC_DIRECT	Extracellular region	34	3.3E-11	3.6	5.8E-9
GOTERM_CC_DIRECT	Extracellular exosome	29	8.9E-8	3.0	7.9E-6
GOTERM_CC_DIRECT	Extracellular space	26	5.0E-7	3.0	3.0E-5
GOTERM_CC_DIRECT	Endoplasmic reticulum-Golgi intermediate compartment membrane	6	3.0E-5	16.6	1.3E-3
GOTERM_CC_DIRECT	Endoplasmic reticulum lumen	9	5.5E-5	6.7	1.9E-3
GOTERM_MF_DIRECT	Serin-type endopeptidase activity	8	2.4E-5	9.2	5.7E-3
GOTERM_MF_DIRECT	Heparan sulfate proteoglycan binding	4	8.6E-5	45.4	1.0E-2
GOTERM_MF_DIRECT	Heparin binding	7	1.7E-4	8.4	1.4E-2
GOTERM_MF_DIRECT	Extracellular matrix structural constituent	6	4.5E-4	9.3	2.6E-2
GOTERM_MF_DIRECT	Protein tyrosine/threonine phosphatase activity	3	9.1E-4	64.7	3.5E-2
*Downregulated genes top 5 enriched GO terms*					
GOTERM_BP_DIRECT	Bicarbonate transport	3	3.5E-3	33.3	1.0E0
GOTERM_BP_DIRECT	Cell adhesion	6	1.4E-2	4.1	1.0E0
GOTERM_BP_DIRECT	Cellular oxidant detoxification	3	1.6E-2	15.4	1.0E0
GOTERM_BP_DIRECT	Receptor-mediated endocytosis	3	1.7E-2	15.0	1.0E0
GOTERM_BP_DIRECT	Blood coagulation	3	2.3E-2	12.6	1.0E0
GOTERM_CC_DIRECT	Receptor complex	7	1.9E-5	12.4	2.1E-3
GOTERM_CC_DIRECT	Plasma membrane	28	5.0E-5	2.0	2.8E-3
GOTERM_CC_DIRECT	Apical plasma membrane	7	4.9E-4	2.1	1.4E-2
GOTERM_CC_DIRECT	Brush border membrane	4	5.1E-4	25.2	1.4E-2
GOTERM_CC_DIRECT	Membrane	19	2.2E-3	2.1	4.9E-2
GOTERM_MF_DIRECT	Fatty acid binding	3	4.2E-3	30.4	3.0E-1
GOTERM_MF_DIRECT	Peroxidase activity	3	4.4E-3	29.6	3.0E-1
GOTERM_MF_DIRECT	Growth factor binding	3	5.2E-3	27.4	3.0E-1
GOTERM_MF_DIRECT	Cytokine binding	3	7.1E-3	23.3	3.0E-1
GOTERM_MF_DIRECT	RNA polymerase II Sequence-specific DNA binding TF binding	4	1.4E-2	7.8	4.7E-1

**Table 4:** Pathway analysis results of common DEGs between four studied datasets.

Pathway	Enrichment FDR	Fold Enrichment	Pathway Genes	genes	Genes
***Pathways of up-regulated DEGs***					
**Path: hsa04512** ECM-receptor interaction	0.0001	18.14	88	6	COMP, FN1, ITGA2, LAMB3, SDC4, SPP1
**Path: hsa01523** Antifolate resistance	0.047	17.73	30	2	TYMS, ABCC3
**Path: hsa04610** Complement and coagulation cascades	0.006	12.52	85	4	CD55, CFI, PLAU, PROS1
**Path: hsa05222** Small cell lung cancer	0.006	11.56	92	4	FN1, ITGA2, LAMB3, RXRG
**Path: hsa05223** Non-small cell lung cancer	0.028	11.08	72	3	MET, RXRG, TGFA
**Path: hsa04115** p53 signaling pathway	0.028	10.93	73	3	SFN, BID, ZMAT3
**Path: hsa04514** Cell adhesion molecules	0.006	8.52	156	5	CDH3, NRCAM, SDC4, CLDN10, CLDN1
**Path: hsa05202** Transcriptional misregulation in cancer	0.003	8.27	193	6	DUSP6, ETV5, MET, PLAU, RXRG, HMGA2
**Path: hsa05215** Prostate cancer	0.047	8.22	97	3	ETV, PLAU, TGFA
**Path: hsa04510** Focal adhesion	0.003	7.98	200	6	COMP, FN1, ITGA2, LAMB3, MET, SPP1
**Path: hsa05205** Proteoglycans in cancer	0.003	7.90	202	6	FN1, ITGA2, MET, PLAU, SDC4, TIAM1
**Path: hsa05206** MicroRNAs in cancer	0.031	6.61	161	4	CYP1B1, MET, PLAU, HMGA2
**Path: hsa04151** PI3K-Akt signaling pathway	0.006	5.26	354	7	COMP, FN1, ITGA2, LAMB3, MET, SPP1, TGFA
**Path: hsa05165** Human papillomavirus infection	0.020	4.82	331	6	COMP, FN1, HEY2, ITGA2, LAMB3, SPP1
**Path: hsa04010** MAPK signaling pathway	0.046	4.52	294	5	DUSP4, DUSP5, DUSP6, MET, TGFA
**Path: hsa05200** Pathways in cancer	0.012	4.015	530	8	FN1, HEY2, ITGA2, LAMB3, MET, RXRG, BID, TGFA
***Pathways of down-regulated DEGs***					
**Path: hsa00350** Tyrosine metabolism	0.006	36.66	36	3	ADH1B, HGD, AOX1
**Path: hsa04630** JAK-STAT signaling pathway	0.022	10.86	162	4	FHL1, GHR, AOX1, LIFR

### 3.4 Exploration of hub genes

**Fig. 5:**
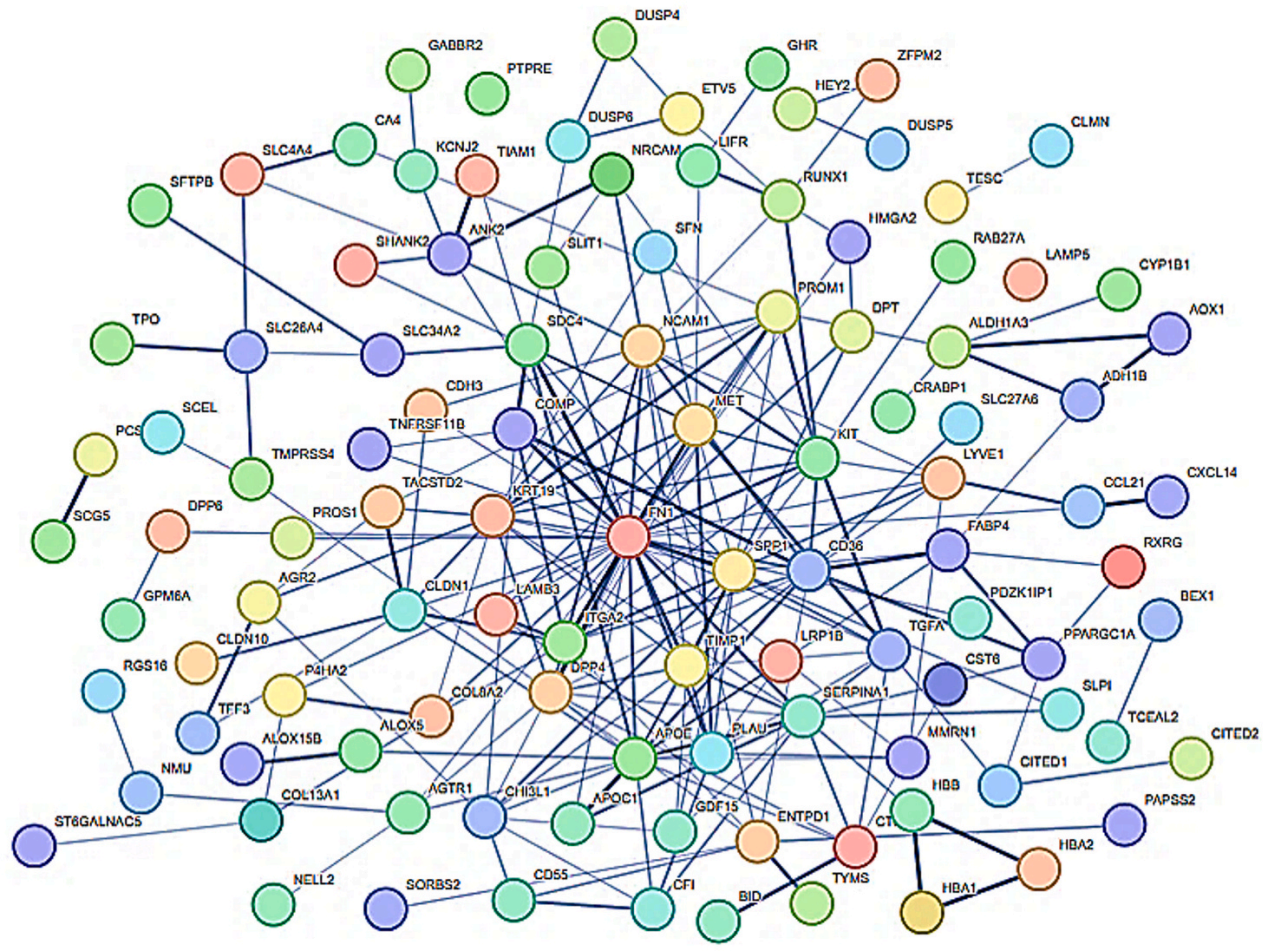
PPI network analysis of common DEGs. The network was generated via the STRING database to elucidate the functional relationships between the intersectional DEGs. Nodes indicate specific genes, while edges represent the documented protein-protein interactions.

**Fig. 6:**
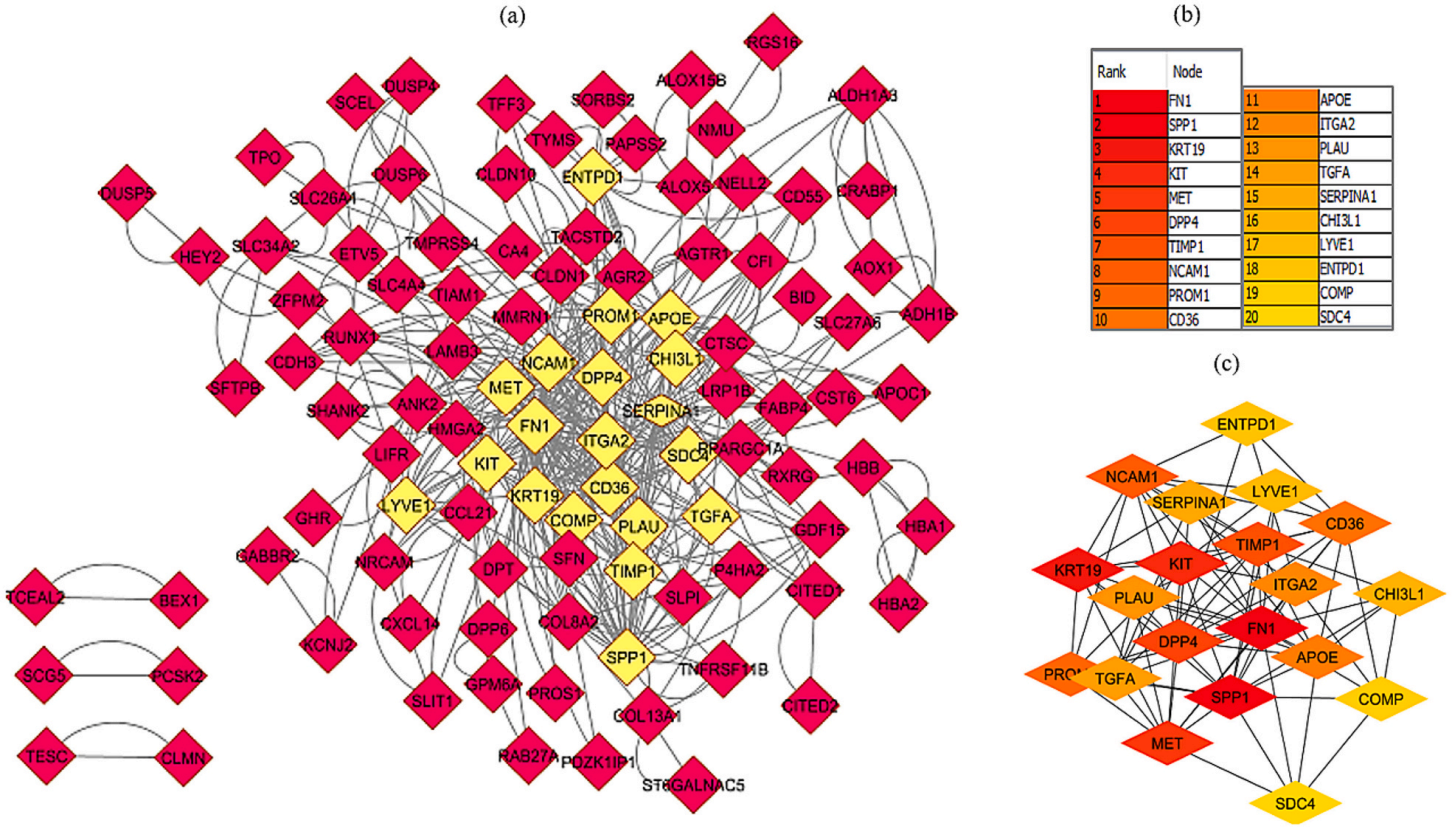
Protein-protein interaction (PPI) network of common DEGs. The nodes represent proteins, and the edges represent functional interactions. Node size and colour intensity correspond to the degree of connectivity (centrality) as calculated by the CytoHubba plugin. The top 20 hub genes are highlighted in red. The network was constructed using the STRING database with a confidence score > 0.4 (a) Visualization of PPI network of all DEGs with 20 genes with the highest degree scores; yellow diamond: top 20 hub genes. (c) Interaction of 20 hub genes based on CytoHubba; darker colour diamonds: higher degree score. (For interpretation of the references to colour in this figure legend, the reader is referred to the web version of this article.)

**Fig. 7:**
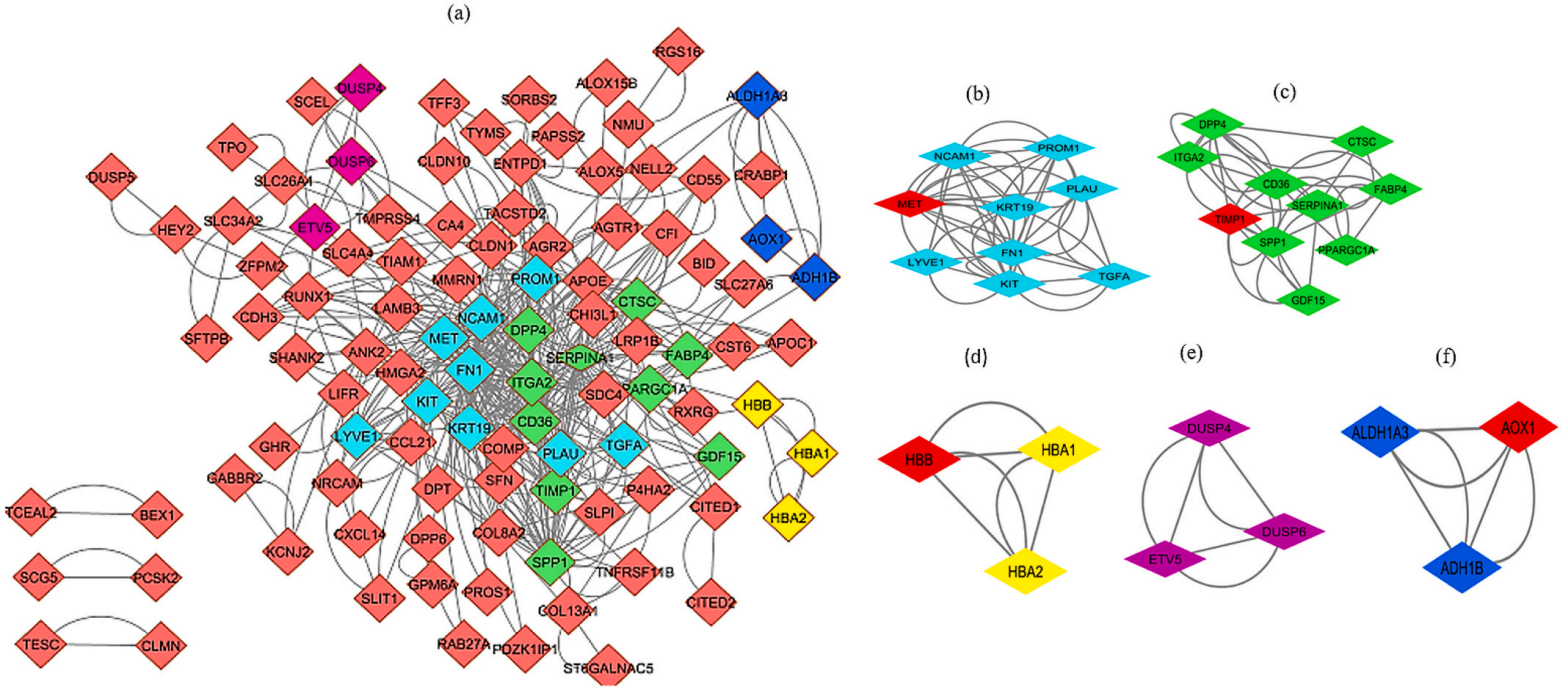
Five significant clusters related to the PPI network of papillary thyroid carcinoma and their properties. (a) PPI network of integrated DEGs; bright blue nodes (cluster1); green nodes (cluster 2); yellow nodes (cluster3); purple nodes (cluster 4); and dark blue nodes (cluster5). (b) Cluster number 1 (nodes: 9; edges: 46; seed node: MET). (c) cluster number 2 (nodes: 10; edges: 36; seed node: TIMP1). (d) cluster number 3 (nodes: 3; edges: 6; seed node: HBB). (e) cluster number 4 (nodes: 3; edges: 6). (f) cluster number 5 (nodes: 3; edges: 6; seed node: AOX1). (For interpretation of the references to colour in this figure legend, the reader is referred to the web version of this article.)

Using the STRING as a biological database, a protein interaction network was created with 148 nodes and 234 edges (Fig. 5). Colored nodes show the queries, and edges represent protein-protein associations. Then, the constructed network was entered into the Cytoscape for a topological analysis of the network. The top 20 hubs (including FN1, KRT19, APOE, SPP1, DPP4, NCAM1, MET, CD36, KIT, SERPINA1, TIMP1, ITGA2, PROM1, PLAU, CHI3L1, TGFA, CLDN1, SDC4, ENTPD1, LYVE1) were selected based on high connectivity (node degree) (Table 5). The top selected 20 hubs were further analyzed by CytoHubba (Fig. 6). Protein clusters of our network were known by the MCODE algorithm in Cytoscape. In this algorithm, all nodes (genes) are weighted using neighborhood density and identified highly connected regions. In this study, MCODE found 5 sub-networks that are presented in Fig.7. These complexes (clusters) help to discover candidate critical genes (or potential biomarkers) for PTC disorder. Subnetworks were ranked based on their size. In every cluster, seed nodes were recognized. The seed represents the highest-weighted node in a cluster.

**Table 5:** Top 20 hub genes with high node degree:

Name	Betweenness Centrality	Closeness Centrality	Degree	Cluster number
FN1	0.36	0.50	34	1
KRT19	0.04	0.41	14	1
APOE	0.13	0.43	19	–
SPP1	0.07	0.43	19	2
DPP4	0.11	0.43	14	2
NCAM1	0.08	0.42	14	1
MET	0.07	0.42	14	1
CD36	0.05	0.41	13	2
KIT	0.06	0.41	13	1
SERPINA1	0.07	0.4	12	2
TIMP1	0.01	0.40	12	2
ITGA2	0.02	0.408	12	2
PROM1	0.09	0.40	10	1
PLAU	0.01	0.37	10	1
CHI3L1	0.02	0.37	9	–
TGFA	0.04	0.39	9	1
CLDN1	0.04	0.37	8	–
SDC4	0.09	0.38	8	–
ENTPD1	0.06	0.35	8	–
LYVE1	0.005	0.37	7	1

### 3.5 Survival analysis for identified hub genes

**Fig. 8:**
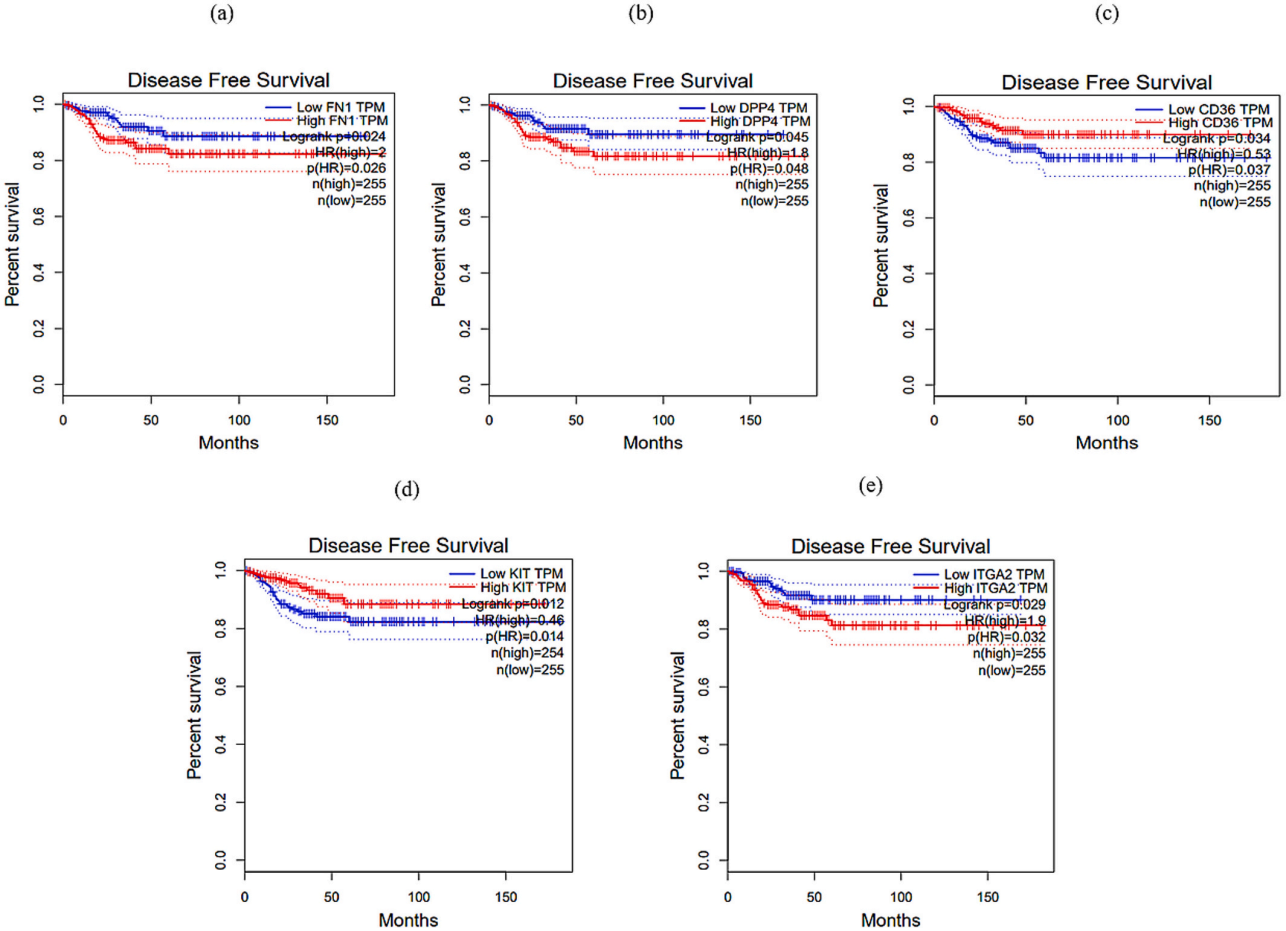
Survival curves for the top five hub genes (FN1, DPP4, CD36, KIT, and ITGA2) in PTC patients. Patients were stratified into high-expression and low-expression groups based on the median expression level. The log-rank test was used to determine statistical significance. P-values <0.05 are considered statistically significant: (a) FN1. (b) DPP4. (c) CD36. (d) KIT. (e) ITGA2.

To decipher the prognostic valence of the potential 20 hubs, Disease-Free Survival (DFS) analysis was done in GEPIA (Fig. 8). In thyroid cancer, DFS serves as a critical prognostic indicator, reflecting the ability of a therapeutic intervention to prevent disease recurrence and maintain long-term remission. We observed that the DFS was less in the high expression groups of FN1 (*p* = 0.024), DPP4 (*p* = 0.045), and ITGA2 (*p* = 0.029) compared with their low expression groups. The low CD36, and KIT expression group had lower DFS than the high CD36 (*p* = 0.034) and KIT (0.012); As presented in Fig. 8, the rest of the hubs (15 genes) were not significantly related to the PTC prognosis (*p* > 0.05). Furthermore, we selected five key genes (including FN1, DPP4, CD36, KIT, and ITGA2) related to the prognosis of PTC.

### 3.6 Verification of hub genes in TCGA

**Fig. 9:**
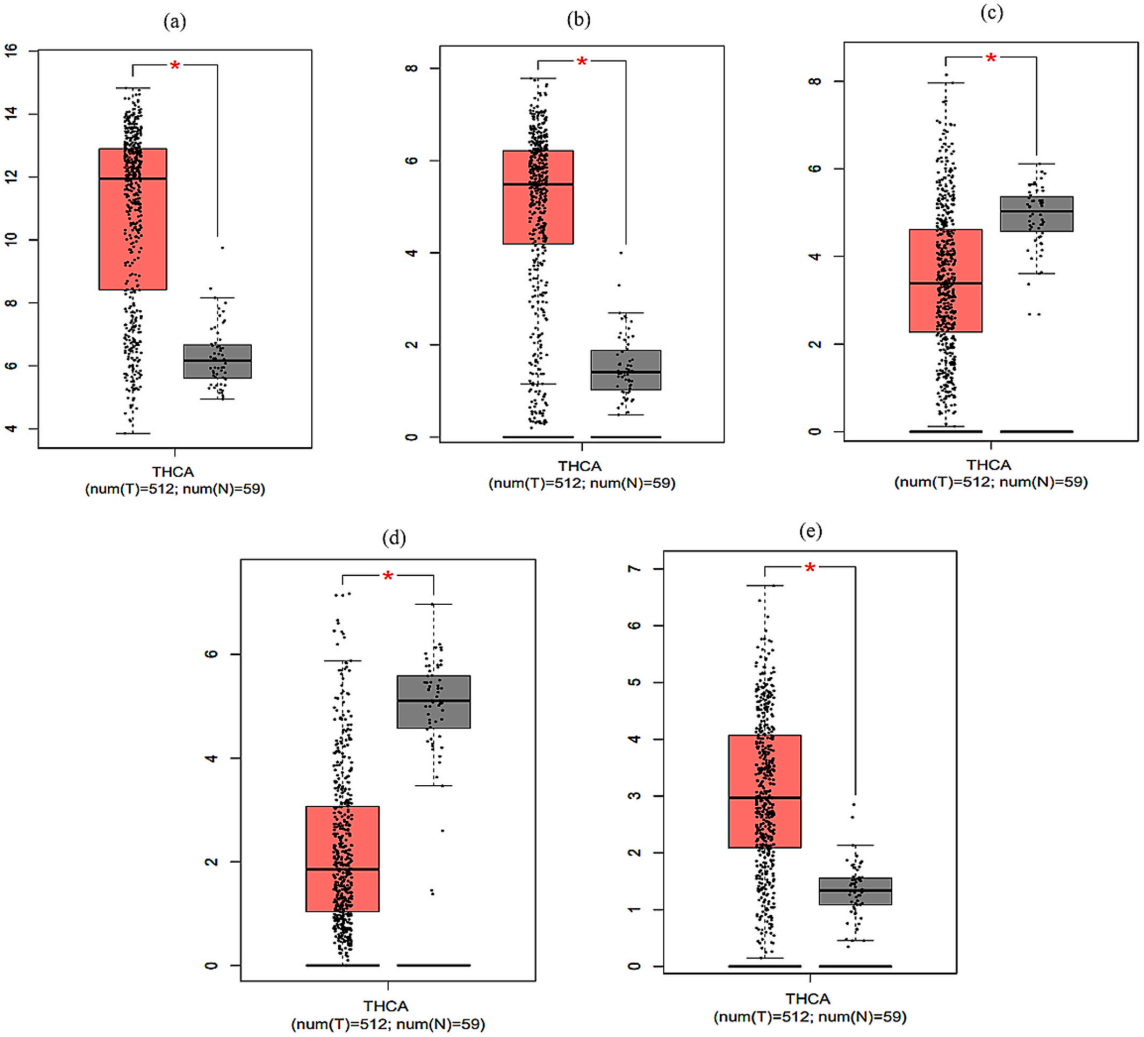
Validation of key gene expression levels via mRNA quantification. Boxplots illustrate the mRNA expression profiles of selected key genes across. (a) FN1. (b) DPP4. (c) CD36. (d) KIT. (e) ITGA2.

Verification of five key genes was conducted by GEPIA in the TCGA database. The findings demonstrated that the 5 key genes had differences in expression levels in normal tissue compared to thyroid cancer tissue samples (Fig. 9).

### 3.7 Interaction network of miRNA-hub gene

**Fig. 10:**
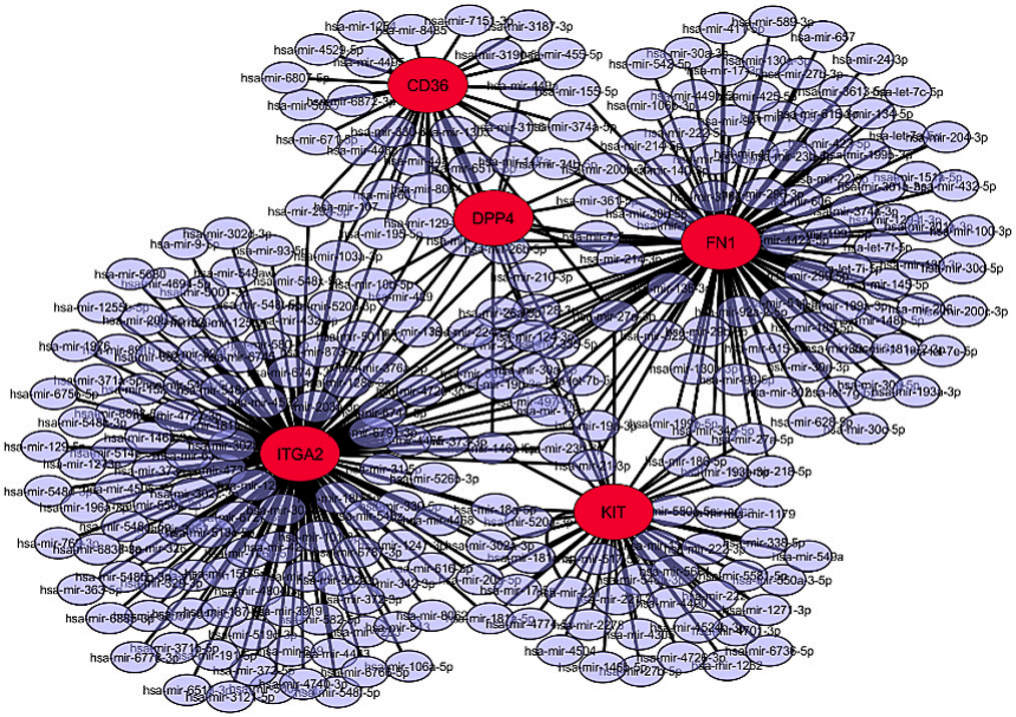
MiRNA-key gene regulatory network: The constructed network illustrates the potential regulatory interactions between miRNAs and key target genes identified in this study. Blue nodes represent miRNAs, and red nodes represent key genes. The edges connecting the nodes indicate the predicted regulatory relationships. (For interpretation of the references to colour in this figure legend, the reader is referred to the web version of this article.)

The miRNet was utilized to detect miRNAs targeting key genes. The miRNA-key genes interaction network was visualized by Cytoscape software. Results showed that has-mir-124-3p interacted with all the five key genes (Fig. 10), which could play a major role in PTC prognosis (Table 6).

**Table 6:** miRNAs list that target key genes.

miRNAs	Target key genes
has-mir-124-3p	FN1, DPP4, CD36, KIT, ITGA2
has-mir-335-5p	FN1, CD36, KIT, ITGA2
has-129-2-3p	FN1, DPP4, CD36, ITGA2
has-mir-16-5p	FN1, CD36, ITGA2
has-mir-19a-3p	FN1, KIT, ITGA2
has-mir-27a-3p	FN1, CD36, KIT
has-mir-21-3p	FN1, KIT, ITGA2
has-mir-146a-5p	DPP4, KIT, ITGA2

### 3.8 Immunohistochemical analysis

**Fig. 11:**
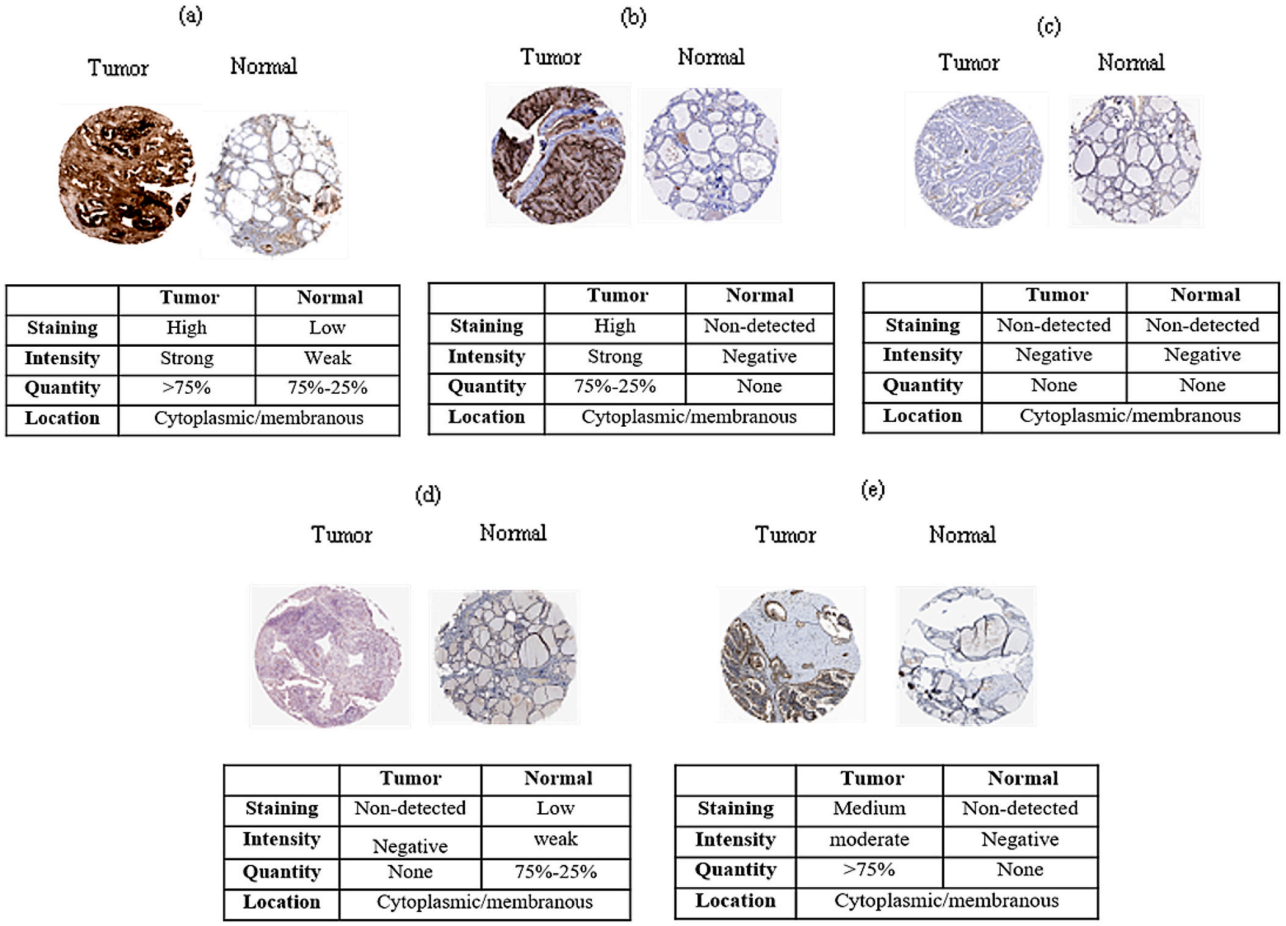
Immunohistochemical analysis of selected hub genes in thyroid tissues. Representative IHC images showing the expression patterns of (a) FN1, (b) DPP4, (c) CD36, (d) KIT, and (e) ITGA2 in papillary thyroid carcinoma (PTC) tissues compared to normal thyroid tissues. Quantification of protein expression levels is based on IHC staining intensity and proportion of positive cells.

Immunohistochemistry (IHC) analysis based on the Human Protein Atlas (HPA) indicated that FN1, DPP4, and ITGA2 were overexpressed in TC tissues, whereas KIT was downregulated in cancerous samples compared with normal tissues (Fig. 11). These results verified our data. Interestingly, no significant dysregulation was found in the level of CD36 between cancer and normal samples. However, we did not observe the relationship between CD36 and thyroid cancer in this analysis. Further experimental data were required to validate this point.

### 3.9 Results of molecular docking

Virtual screening and docking are vital features of molecular modelling methodology that focuses on studying the interactions between proteins and enzymes with ligands.^15^ The three-dimensional (3D) structure of hub genes (druggable genes), including CD36 (PDB ID: 5LGD),^18^ DPP4 (PDB ID: 4A5S),^19^ FN1 (PDB ID: 2CG7),^20^ ITGA2 (PDB ID: 1AOX),^21^ and KIT (PDB ID: 3G0E)^22^ were downloaded from the PDB: https://www.rcsb.org/n.^13^ Based on the results of virtual screening, from 1615 FDA drugs, the top 5 ligands (drugs) with the highest binding affinity (BA) to each one of the five key genes (proteins) were derived for further investigation. The grid box for CD36, DPP4, FN1, ITGA2, and KIT was set to a cubic box mentioned in Table 7, and 0.375 Å in each dimension as a grid point spacing. The docking simulations were performed with 200 runs, and the total number of energy evaluations was set to 25,000,000 (number of evaluations).

**Table 7:** The grid box and grid center dimensions of targets (proteins) for each drug.

Target	Grid Box (Å^3^)	Grid center
CD36	90 × 90 × 100	−41.83× −28.05 × 27.51
DPP4	80 × 80 × 80	16.75 × 33.48 × 56.47
FN1	60 × 70 × 60	7.38 × 11.10 × 13.47
ITGA2	60 × 70 × 60	0.13 × 5.53 × −2.74
KIT	70 × 70 × 70	37.51 × −8.27 × −78.47

**Fig. 12:**
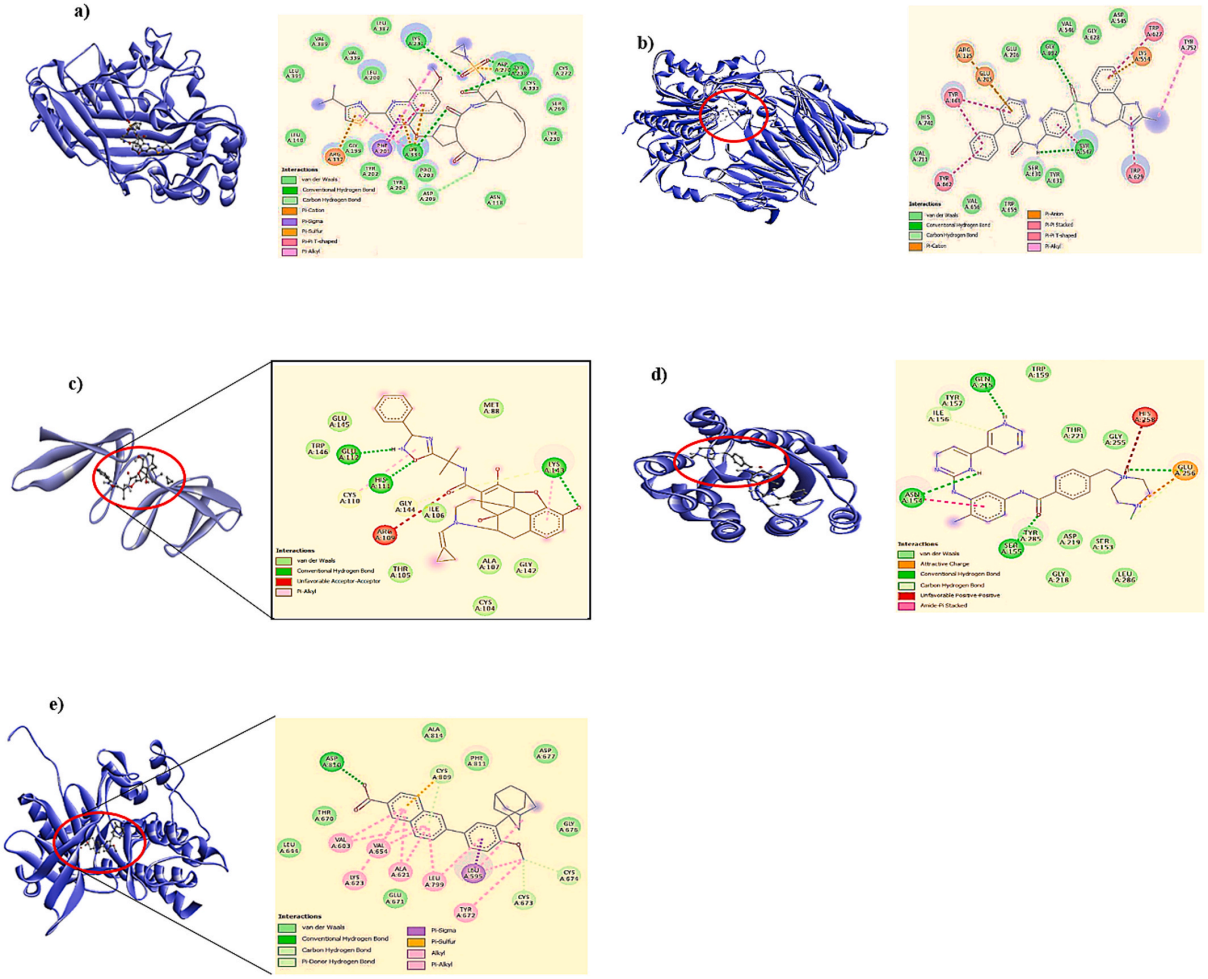
The 3D structure of as a ribbon structure and 2D scheme of binging site. The complexes of a) CD36 with Olysio, b) DPP4 with Gabapentin, c) FN1 with Naldemedine, d) ITGA2 with Imatinib and e) KIT with Enzacamene. The H-bond interaction is depicted with green dotted line. (For interpretation of the references to colour in this figure legend, the reader is referred to the web version of this article.)

In this stage, ligand selection was done by the following steps: the BA, the inhibition constant (KI), the number of hydrogen and Van der Waals interactions, and total carbon‑hydrogen bonds (Table 8). We found the highest BA at −11.88 kcal/mol for CD36 with Olysio, −11.50 kcal/mol for DPP4 with gabapentin, −10.37 kcal/mol for FN1 with Naldemedine, −9.33 kcal/mol for ITGA2 with imatinib and − 10.80 kcal/mol for KIT with Enzacamene by using the auto dock. As seen in Table 8, the results demonstrated that the hydrogen bonds and van der Waals interaction interaction contributed significantly to the stability of these complexes. Redocking of the co-crystallized ligands in the binding sites of CD36, DPP4, and KIT reproduced the crystallographic poses with RMSD values of 1.407, 0.434, and 1.378 Å, respectively, demonstrating excellent agreement between the docked and reference conformations. The corresponding binding energies were − 5.23, −14.42, and − 8.50 kcal/mol, supporting a stable predicted interaction. FN1 (PDB ID: 2CG7) and ITGA2 (PDB ID: 1AOX) are ligand-free and in their apo forms. Therefore, they were not agreeable to direct redocking validation. Consequently, docking for FN1 and ITGA2 was performed without a co-crystallized ligand-based positive control. To additionally confirm the specificity of the docking results, a negative control was applied using HSA as an unrelated non-target protein. Blind docking was achieved for the top five energy drugs by parameters identical to those applied for the target proteins. The binding affinities of Olysio, Gabapentin, Naldemedine, Imatinib, and Enzacamene to HSA were − 7.5, − 4.5, − 10.75, − 5.6, and − 5.9 kcal/mol, respectively. These values were consistently weaker (less negative) than the corresponding affinities for the therapeutic targets, with an average difference of approximately 4.5 kcal/mol. Notably, Naldemedine exhibited a relatively higher affinity for HSA (−10.75 kcal/mol), which may be attributed to its known high plasma protein-binding properties. Nevertheless, the overall trend confirms that the observed binding to CD36, DPP4, FN1, ITGA2, and KIT is target-specific and not merely a consequence of general hydrophobic or electrostatic interactions with any protein surface. Taken together, the positive and negative control results substantiate the accuracy and specificity of the docking method employed in this study. To better illustrate the ligand-protein interactions, as shown in Fig. 12, the optimal 3D and 2D representations of the ligand-receptor interaction were depicted. To evaluate the clinical feasibility of drug repurposing, we transitioned from a single-target analysis to a multi-target perspective. We implemented a scoring system to rank drugs based on their ability to interact with multiple key proteins simultaneously. As summarized in Table 9, Nilotinib was identified as the most promising multi-target candidate, exhibiting a high binding affinity for CD36, DPP4, and ITGA2.

**Table 8:** The characteristics of vina and AutoDock binding energies for 5 top ligands with the highest binding affinity (kcal/mol), Inhibition constant KI (μM), Number of Hydrogen bonds, van der Waals and carbon hydrogen bonds (CHB) interactions.

Proteins	Drug name	ZINC Comp. IDs	Docking results		K_I_ (μM)	No. of H bonds	No. of VDW	No. of CHB
			Vina	AutoDock				
CD36	Nilotinib	6,716,957	−11.6	−10.05	4.27 × 10^−2^	2	12	2
	Sqv	26,985,532	−11.1	−11.00	8.65 × 10^−3^	5	12	2
	**Olysio**	164,760,874	−11.1	−11.88	1.96 × 10^−3^	4	16	1
	Midostaurin	100,013,130	−11	−9.67	8.11 × 10^−2^	–	13	1
	Fosaprepitant	3,939,013	−10.9	−7.95	1.49	3	7	3
DPP4	Dihydroergotamine	3,978,005	−12.2	−9.84	6.21 × 10^−2^	2	12	1
	**Gabapentin**	13,973,998	−11.9	−11.50	3.71 × 10^−3^	2	8	2
	Naldemedine	100,378,061	−11.7	−10.44	2.21 × 10^−2^	3	13	1
	Natamycin	169,621,220	−11.6	−10.69	1.47 × 10^−2^	4	13	1
	Nilotinib	6,716,957	−11.6	−10.14	3.72 × 10^−2^	3	12	–
FN1	Dihydroergotamine	3,978,005	−9.3	−9.93	5.27 × 10^−2^	2	8	3
	**Naldemedine**	100,378,061	−9.2	−10.37	2.49 × 10^−2^	3	8	2
	Daunorubicin	3,917,708	−9	−9.82	6.31 × 10^−2^	6	3	1
	Midostaurin	100,013,130	−9	−8.93	2.86 × 10^−1^	1	6	2
	Ergotamine	52,955,754	−9	−9.47	1.14 × 10^−1^	1	6	2
ITGA2	Accolate	896,717	−7.7	−8.21	9.60 × 10^−1^	3	8	1
	Nilotinib	6,716,957	−7.6	−8.05	1.26	2	9	3
	**Visudyne**	150,338,699	−7.3	−8.32	7.94 × 10^−1^	5	6	1
	Vilazodone	1,542,113	−7.5	−8.22	9.47 × 10^−1^	5	8	–
	Imatinib	19,632,618	−7.2	−9.33	1.45 × 10^−1^	4	10	–
KIT	**Enzacamene**	2,008,310	−10.7	−10.80	1.20 × 10^−2^	1	7	3
	Lumacaftor	64,033,452	−10.5	−9.43	1.22 × 10^−1^	3	14	1
	Erismodegib	68,202,099	−10.4	−8.35	5.37 × 10^−1^	2	7	2
	Gabapentin	13,973,998	−10.3	−10.17	3.54 × 10^−2^	–	12	–
	Eht0201	1,530,776	−10	−9.70	7.71 × 10^−2^	3	10	–

**Table 9:** Ranking of potential drug repurposing candidates based on their multi-target potential score (MPS) and binding affinities across key hub proteins.

Rank	Drug Name	Targets Covered	Average Binding Affinity (Vina)	Multi-Target Potential
1	Nilotinib	CD36, DPP4, ITGA2	−11.6	Very High (Triple Target)
2	Gabapentin	DPP4, KIT	−11.1	High (Double Target)
3	Naldemedine	DPP4, FN1	−10.45	High (Double Target)
4	Midostaurin	CD36, FN1	−11.0	High (Double Target)
5	Dihydroergotamine	DPP4, FN1	−10.75	High (Double Target)

## 4 Discussion

Although thyroid cancer is associated with less mortality, it has increased more than expected in recent years. Therefore, the identification of novel diagnostic, prognostic, and therapeutic biomarkers with high accuracy and precision is more necessary. In addition, the detection of new molecular-based targets can reduce the financial burden imposed on individuals and society. A primary objective of this work was to discover a panel of new diagnostic and prognostic biomarkers related to the overall survival in individuals with PTC by analysis of the PPI network. In the current study, the bioinformatics method and protein network analysis were used to analyze the selected datasets (including GSE58545, GSE3467, GSE29265, and GSE60542) from GEO. We used LogFC ≥1.5 as an index to detect DEGs in each dataset and then integrated all of them. The LogFC values show the level of dysregulation of the DEGs.^23^ Thus, we were assumed that the DEGs with this LogFC level would be the genes broadly contributing to the molecular mechanism of PTC, and gene ontology and pathway analyses were carried out on the integrated DEGs. In the present study, GO analysis revealed that DEGs were related to cell adhesion (P: 2.0E-4), and cell-cell adhesion (P: 4.6E-1). There is evidence that many of the cell adhesion molecules (CAMs) play roles as tumor suppressors, and alterations in these molecules are more frequently observed during the development of cancer.^24^ It has been manifested that dysregulated expression of CAM in several malignant cancers such as thyroid cancer is broadly linked to poorer prognosis, distant metastases, and increased primary invasion. Based on these findings, it suggests that individual CAM patterns could potentially serve as biomarkers for diagnosing thyroid cancers in the future.^25^, ^26^ Rabi et al. investigated the clinical application of these molecules in the controlling of thyroid nodules. They also reported that L-selectin (SELL), ICAM1 genes, L-selectin, and LFA-1 protein expression can help verify malignancy and collaborate in the histological featurization of follicular patterned lesions; however, we couldn't establish a connection between patient outcomes with these CAMs. In addition, literature has reported that alterations in cell-cell adhesion can promote metastasis of cancer cells.^27^ For instance, PTC with strong expression of neural cell adhesion molecule (NCAM) was more likely to have an extracapsular invasion.^28^The KEGG pathway analysis of our work showed that the up- and downregulated genes were primarily enriched in ECM-receptor interaction and tyrosine metabolism. It was observed that the interaction between cancer cells and ECM proteins serves a vital affect in the invasion, as well as the metastasis of tumors.^29^ In addition, ECM-receptor interaction plays an essential role in tumor degradation, tumor adhesion as well as, and tumor hyperplasia.^30^ Several bioinformatics analyses also reported ECM-receptor interaction as the main pathway involved in thyroid carcinoma.^31^, ^32^Additionally, the enrichment of the **ECM-receptor interaction** pathway in our upregulated DEGs is highly congruent with recent findings regarding the remodeling of the tumor microenvironment in papillary thyroid carcinoma. Recent investigations have demonstrated that alterations in extracellular matrix components are critical drivers of tumor cell invasion and survival.^33^ Our results reinforce this paradigm, suggesting that targeting ECM-related signaling could be a viable therapeutic strategy. In the protein network analysis, we applied the STRING server to construct a network using integrated DEGs (including upregulated and downregulated genes) based on the functional association. We utilized the MCODE algorithm to cluster the whole network into smaller subnetworks. Five subnetworks were obtained. Here, we had seed nodes including MET, TIMP1, HBB, and AOX1 in cluster numbers 1, 2, 3, and 5, respectively (Fig.7). The MET encodes a receptor tyrosine kinase family of proteins. Garcia et al. reported that the TTA1 cell line that was derived from anaplastic thyroid cancer (ATC) expressed MET to a high level. In addition, many studies found a remarkable association between overexpression of MET and a rising risk of metastasis in PTC. In another study, it was reported that normal thyroid cells don't express MET, while its up-regulation was found in thyroid cancer and correlated with undesirable outcomes.^34^ The TIMP1 gene, which belongs to the TIMP family, produces matrix metalloproteinase (MMP) natural inhibitors. Dimitrova et al. investigated TIMP1 expression in fine-needle aspiration (FNA) biopsies from PTC patients in contrast to benign nodules. They reported that the patients with PTC had higher levels of TIMP-1 in serum than the benign nodules.^35^ In another study, RT-qPCR was used to determine the mRNA relative expression of the TIMP1 gene in the FNA of benign and malignant thyroid nodules and the results showed the significant up-regulation of its transcripts in malignant nodules. However, TIMP1 could be a promising biomarker for thyroid nodule monitoring.^36^ Hemoglobin subunit beta (HBB) was detected as another seed gene in our analysis. Onda et al. evaluated the gene expression profile of 11 ATC cell lines and reported significantly reduced expression of the HBB in the ACL.^37^ This result offered the possibility of the HBB as a new potential tumor suppressor. Recently, Nan et al. presented HBB as a seed node in the analysis of miRNA-mRNA regulatory networks in PTC.^38^ AOX1 was detected as the latest seed gene in our analysis. Prognostic analysis demonstrated that aldehyde oxidase 1 (AOX1), a low level of *AOX1* was markedly associated with poor survival in PTC patients.^39^ More recently, Wang et al. observed the overexpression of AOX1 in normal samples compared to PTC samples.^40^

In addition, we used Cytoscape to select the top 20 hub genes according to the connectivity degree of the genes. Finally, five key genes associated with PTC prognosis were selected. Among the identified key genes, FN1 and ITGA2 were upregulated genes, while DPP4, CD36, and KIT were downregulated ones. Additionally, gene expression of these five key genes was validated by GEPIA, and HPA was applied to verification of gene expression in protein levels. Our prognostic analysis highlights a critical link between the expression of five key hubs and patient outcomes in PTC. Specifically, the significant correlation between the high expression of FN1, DPP4, ITGA2, CD36, and KIT with reduced DFS suggests that these genes act as potent drivers of disease recurrence. These findings identify these five genes not only as robust prognostic biomarkers but also as high-priority therapeutic targets for preventing clinical relapse. Our identification of FN1 and DPP4 as central hub genes aligns with some transcriptomic studies in thyroid malignancies. For instance, Li et al. reported that the dysregulation of FN1 is closely associated with melanoma metastasis and promotes melanoma proliferation and metastasis by inhibiting apoptosis and regulating Epithelial to mesenchymal transition (EMT),^41^ which is consistent with our finding that FN1 overexpression correlates with decreased DFS. Similarly, the role of DPP4 in regulating the inflammatory process has been highlighted in recent studies on thyroid diseases,^42^ supporting our prediction of its involvement in PTC progression. In addition, Cheng et al. reported that the DPP4/CTNNB1/MET is an oncogenic signature that is overexpressed in papillary thyroid cancer and associated with disease progression, distant metastasis, treatment resistance, immuno-evasive phenotypes, and poor clinical outcomes.^43^ Additionally, in the current study, molecular docking and virtual screening identified several FDA-approved drugs (Olysio, Gabapentin, Naldemedine, Imatinib, and Enzacamene) as potential therapeutic candidates for PTC, targeting our identified hub genes.^44^ Although these drugs were originally developed for other clinical indications, their identification in our study relies on the principle of ‘Drug Repurposing’,^45^, ^46^ where computational binding affinity suggests a potential for targeting the proteins involved in thyroid cancer pathogenesis.

Fibronectin (FN1) with the maximum degree of connection was detected as both a hub and a key gene in our analysis. FN1 is a glycoprotein that is generated by fibroblasts and tumor cells. It affects blood coagulation, proliferation, differentiation, and metastasis, among other cell adhesion and migration processes.^47^ Tumor suppression is a function of cancerous FN, but it is associated with a poor prognosis. Furthermore, the tumor's growth is aided by the FN matrix that is deposited in the tumor microenvironments (TMEs).^47^ Pecce et al. have demonstrated that FN1 in an overexpressed state is a main determinant of aggressiveness in thyroid cancer. Their results indicated that the FN1 mRNA level was higher in tumor vs. non-tumor tissue and also in aggressive samples.^48^ In another investigation, Xia et al. reported that migration and invasion of PTC are promoted by FN1; however, it is a valuable diagnostic marker to predict lymph node metastasis (LNM) of PTC.^49^ In the current analysis, FN1 (a hub and key gene) was expressed at a higher level in tumors than in the adjacent normal tissues. Additional verification of the relationship between FN1 and PTC development may discover novel targets for PTC treatment. Based on our results, Naldemedine was identified as a high affinity drug for FN1 protein among five top 5 binding affinity drugs. Opioid-induced constipation (OIC) is treated with Naldemedine, an opioid antagonist. Additionally, it was found that Naldemedine enhanced the quality of life for individuals with OIC and cancer and effectively and promptly relieved OIC symptoms.

The dipeptidyl peptidase 4 (DPP4) is an intrinsic transmembrane glycoprotein and a serine exopeptidase that is observed in various tumors, but the link between DPP4 and thyroid carcinoma (THCA) is not completely identified. Gao et al. investigated the DPP4 expression in THCA and assessed the association between the expression of DPP4 and antitumor immunity and the prognosis of thyroid cancer.^50^ Finally, they concluded that DPP4 may be a useful target gene for the immunotherapy strategy and a possible biological marker for THCA prognosis.^50^ It has been shown that the variation in the serum levels of DPP4 does not involve in early detection and prognosis of medullary thyroid cancer (MTC).^51^ Paradoxically, Lee et al. discovered a relationship between metastasis and an aggressive condition in PTC and increased DPP4 expression; however, elucidation of DPP4 might be a therapeutic option for thyroid cancer.^52^ This inconsistency in the literature concerning DPP4 expression could be attributed to several factors, such as differences in experimental methodologies, variations in tissue sampling techniques, or the specific histological subtypes of thyroid cancer that were examined. Additionally, the expression profile of DPP4 may vary based on the metabolic state and the progression stage of the tumor. In our research, the identified downregulation implies that DPP4 may have a distinct role in the metabolic reprogramming of PTC cells, potentially affecting the signaling pathways that regulate tumor survival and intercellular interactions.

Molecular docking results indicate that gabapentin can bind to the DPP4 protein's active site with a binding energy of −11.50 kcal/mol (Table 8). The FDA approved the anticonvulsant medication gabapentin in 1993, and it has been sold in the US in generic form since 2004. Gabapentin was originally applied as a muscle relaxant and anticonvulsant. However, it was then found that this drug has potential as an anticonvulsant medication and can be used as an adjunct to more effective anticonvulsant medications.^53^ Gabapentin and Naldemedine were identified through in silico screening due to their potential to modulate signaling pathways critical to cell adhesion and tumor progression, specifically targeting DPP4 and FN1 pathways.^54^ While their primary clinical indications are anticonvulsant and peripheral opioid antagonism, respectively, the principle of drug repurposing suggests that such agents can exert therapeutic effects through novel off-target interactions within oncogenic signaling networks.^45^ Their demonstrated structural affinity for these hub proteins positions them as intriguing candidates for further pharmacological investigation in the context of thyroid cancer.

Cluster of differentiation 36 (CD36) is widely expressed in different types of human tumors, and its expression is concerned with progressive stages, low prognosis, and survival rate.^55^, ^56^ Typically, in cancer cells, CD36 increases fatty acid uptake, alters lipid metabolism, and accelerates cancer cell proliferation, while also facilitating Epithelial-to-Mesenchymal Transition (EMT).^55^ The study of the relationship between CD36 and PTC is very limited, and interestingly, we did find no correlation between CD36 and thyroid cancer in the HPA database. Although our HPA analysis did not reveal a significant correlation between CD36 protein levels and thyroid cancer, it is not unusual in oncology to observe discrepancies between mRNA and protein expression. Nevertheless, our in-silico docking results underscore the functional potential of CD36 as a therapeutic target, indicating that its significance may be more closely associated with its specific binding affinities rather than just its expression levels. According to the results, Olysio can bind to CD36 with high affinity. Consequently, this medication, which has received FDA approval, can serve as suitable ligands to engage with this essential protein and subsequently inhibit it. While Olysio is primarily antiviral, its potential in modulating lipid metabolism-related receptors like CD36 aligns with recent evidence emphasizing the metabolic reprogramming of tumor cells.^57^

The KIT gene produces a receptor tyrosine kinase. This was primarily known as a homolog of the feline sarcoma viral oncogene v-kit and is mostly named proto-oncogene c-Kit. The protein encoded by this gene plays a critical role in the proliferative process, differentiation, migration, and apoptosis of several types of cells. Mutations of this gene are related to some cancers. Hub genes such as KIT were discovered by Li and colleagues as a possible biomarker for thyroid cancer diagnosis and prognosis.^58^ This result is in accordance with the results of our work. Molecular docking of FDA approved compounds with KIT protein showed significant negative binding affinity of Enzacamene. Although Enzacamene conventionally used as a UV-filtering agent, our molecular docking analysis showed a high binding affinity between Enzacamene and the KIT protein. This suggests that Enzacamene might interact with signaling domains of tyrosine kinases, a mechanism that warrants further in vitro investigation to explore its potential as a novel scaffold for kinase inhibition in oncology.^59^

Integrin alpha 2 (ITGA2) is a transmembrane receptor for collagens.^60^ ITGA2 primarily facilitates cell adhesion to the extracellular matrix (ECM).^61^ ITGA2 is upregulated in diverse cancers.^62^, ^63^ Here, we observed that ITGA2 upregulated in PTC tissues and ECM-receptor interaction was the significant pathway that was enriched from upregulated genes. Also, emerging data has indicated that ITGA2 may modulate cell migration, invasion, and metastasis of tumors.^64^ Ropivacaine is a local anesthetic, and recently, several researchers have studied its role in cancers. Accumulating data has reported that Ropivacaine is crucial for preventing tumors like breast, colon, and stomach cancers.^65^, ^66^ However, the role of Ropivacaine has not been completely studied in the PTC progression. Evidence suggests that ITGA2 is a recently detected marker of thyroid malignancy and ITGA2 is strikingly overexpressed in thyroid cancer.^67^ However, inhibition of ITGA2 can quench the progression of several cancers.^68^ In this regard, Qin et al. explored the functional impact of Ropivacaine on papillary thyroid cancer (PTC) cells, assessing its influence on proliferation, invasion, migration, and apoptosis. Their goal was to uncover the underlying mechanisms contributing to the progression of PTC. Their results proved that Ropivacaine could suppress proliferation, invasion, and migration, and impress apoptosis of PTC cells by regulating the expression of ITGA2.^69^ The docking interaction indicated that Imatinib had the highest negative binding energy and with ITGA2 protein. Imatinib, a tyrosine kinase inhibitor, serves as a treatment for leukemias, dermatofibrosarcoma protuberans, systemic mastocytosis, hypereosinophilic syndrome, myelodysplastic/myeloproliferative disease, and gastrointestinal stromal tumors. This result suggests the possibility of designing a high-affinity inhibitor based on Imatinib against ITGA2.

A significant discovery from our research is the recognition of hsa-mir-124-3p as a key regulator within the established network. Although numerous studies concentrate on protein-coding genes, the regulatory function of miRNAs is essential for refining the oncogenic environment. Our investigation reveals that hsa-mir-124-3p acts as a central hub, likely exerting multi-target regulatory influence over five critical hub genes including FN1, DPP4, CD36, KIT, and ITGA2. In various cancers, hsa-mir-124-3p has been identified as a tumor suppressor; its reduced expression may result in the unregulated activation of oncogenes associated with cell cycle progression and metastasis.^70^ In the PTC regard, Sun et al. reported that miR-124-3p inhibits the proliferation and motility, and induces apoptosis and cell cycle arrest in PTC cells.^71^ In addition, the previous studies indicated that the circulating microRNAs such as microRNA 124-3p may be a potential signature for differential diagnosis of thyroid nodules and cancers.^72^, ^73^ Notwithstanding this, the practical use of miR-124 for the early diagnosis and treatment of cancer encounters numerous substantial obstacles, such as enhancing its stability and bioavailability, as well as creating efficient in vivo delivery mechanisms. The ability of hsa-mir-124-3p to coordinate this particular gene module indicates that it could be a primary factor driving the dysregulated phenotype seen in our GEO datasets, positioning it as a high-priority candidate for additional functional validation.

This analysis, while offering considerable strengths, is accompanied by several limitations that require attention. A principal limitation stems from the lack of experimental verification concerning the presented key hub genes and their correlated pathways. The bioinformatic findings provide valuable predictive insights; however, further in vitro and in vivo research is required to establish the definitive biological activity of FN1, DPP4, CD36, KIT, and ITGA2 in the pathogenesis of PTC.

### 4.1 Limitations and future perspective

Despite the valuable insights provided by our study, certain limitations must be acknowledged. First, our findings are primarily based on computational predictions derived from existing GEO datasets and molecular docking simulations. While these methods are highly efficient for identifying candidates, they cannot fully replicate the complex biological environment of a living organism. Therefore, the binding affinities and the multi-targeting potential of the identified drugs, such as Nilotinib, require further validation through in vitro (cell line) and in vivo (animal model) experimental studies to confirm their efficacy and safety profiles. For instance, some studies reported network pharmacology approach for investigating the multi-target mechanisms of compounds in the treatment of various diseases such as cancer.^74^, ^75^, ^76^ Additionally, while we identified has-mir-124-3p as a potential regulator, the intricate multi-layered regulatory network involving mRNA-miRNA-protein interactions is highly dynamic. Our study focuses on a snapshot of these interactions, which may not fully capture the temporal changes in gene expression during PTC progression. Future research should focus on closing the gap between our in-silico discoveries and their clinical applications through several essential steps. First, it is necessary to validate the expression profiles of the hub genes we have identified, along with the regulatory function of has-mir-124-3p, using functional assays such as quantitative real-time PCR (qPCR) and Western Blotting in established PTC cell lines. In addition, to confirm the therapeutic potential of the multi-target drug candidates identified, comprehensive cellular assays, including studies on cell proliferation, migration, invasion, and apoptosis, are required. Finally, it will be crucial to transition from computational predictions to in vivo animal models to assess the pharmacological efficacy, safety, and pharmacokinetic profiles of these drug-repurposing candidates within a complex physiological context. Such empirical validation is vital for advancing precision medicine strategies in the treatment of papillary thyroid carcinoma.

## 5 Conclusion

In the current research, gene profile analysis methods coupled with docking technique investigations were employed to identify possible druggable genes and compounds for the PTC treatment. Our investigation demonstrated that the 5 key genes (including FN1, DPP4, CD36, KIT, and ITGA2) and their associated miRNAs such as has-mir-124-3p that are broadly associated with the tumor's development and progression, indicating that these genes are widely linked to tumor development and progression, suggesting they could be new potential biological markers for prognosis and therapeutic targets in PTC. In this context, the perfect drug-protein interactions of Olysio, Gabapentin, Naldemedine, Imatinib, and Enzacamene can be seen as new therapeutic insights in papillary thyroid carcinoma. Furthermore, our results from this analysis improve understanding of the involved mechanisms in PTC, which can be helpful in the identification of effective biomarkers and, in particular, treatment strategies focusing on key genes and also key regulatory pathways. Nonetheless, additional experimental and clinical evaluations are required to confirm the findings of this study and assess the practical clinical significance of these putative biomarkers.

## Abbreviations

PTC: Papillary Thyroid Carcinoma

TKIs: Tyrosine Kinase Inhibitors

DPP4: Dipeptidylpeptidase 4

FN1: Fibronectin 1

TIMP1: Tissue Inhibitor of Metalloproteinase 1

KEGG: Kyoto Encyclopedia of Genes and Genomes

GO: Gene Ontology

HPA: Human Protein Atlas

BP: Biological Process

CC: Cellular Component

MF: Molecular Function

GO: Gene Ontology

DAVID: Database for Annotation, Visualization and Integrated Discovery

PPI: Protein-Protein Interaction

TMEs: Tumor Microenvironments

CAMs: Cell Adhesion Molecules

SELL: L-selectin

NCAM: Neural Cell Adhesion Molecule

ATC: Anaplastic Thyroid Cancer

MMPs: Matrix Metalloproteinase

HBB: Hemoglobin Subunit Beta

AOX1: Aldehyde Oxidase 1

LNM: Lymph Node Metastasis

MTC: Medullary Thyroid Cancer

TEMs: Tumor Microenvironments

LNM: lymph node metastasis

DPP4: Dipeptidyl Peptidase 4

IGA2: Integrin alpha 2

MTC: Medullary Thyroid Cancer

CD36: Cluster of differentiation 36

BA: Binding Affinity

EMT: Epithelial-to-Mesenchymal Transition

miRNAs: microRNAs

## Availability of data and materials

The data supporting the findings of this study are openly accessible from the GEO, GEO2R, DAVID, STRING, Cytoscape, Network Analyst, GEPIA, TCGA, miRNet, HPA, and AutoDock Vina package in the PyRx program, and BIOVIA Discovery Studio Visualizer platform.

## Clinical trial

This study is not a clinical trial.

## Declaration of generative AI use

In the present study, we didn't use the AI for writing of manuscript and the generation of article structure.

## CRediT authorship contribution statement

**Mehdi Koushki:** Writing – review & editing, Writing – original draft, Validation, Methodology, Formal analysis. **Masoumeh Farahani:** Writing – review & editing, Writing – original draft, Validation, Formal analysis. **Nasrin Amiri-Dashatan:** Writing – review & editing, Writing – original draft, Supervision, Methodology, Funding acquisition, Formal analysis, Conceptualization. **Fatemeh Fateminasab:** Writing – review & editing, Writing – original draft, Formal analysis. **Reza Fadaei:** Writing – review & editing, Writing – original draft, Methodology. **Hossein Chiti:** Writing – review & editing, Writing – original draft, Methodology. **Somayeh Chahkandi:** Writing – review & editing, Writing – original draft. **Lida Perseh:** Writing – review & editing, Writing – original draft.

## Consent for publication

Not applicable.

## Ethics approval and consent to participate

Not applicable.

## Funding

This work was financially supported by grant (A-12-1765-2) from the Deputy of Research, 10.13039/501100008323Zanjan University of Medical Sciences.

## Declaration of competing interest

The authors declare the following financial interests/personal relationships which may be considered as potential competing interests: Nasrin Amiri-Dashatan reports financial support, administrative support, statistical analysis, and writing assistance were provided by Zanjan University of Medical Sciences. If there are other authors, they declare that they have no known competing financial interests or personal relationships that could have appeared to influence the work reported in this paper.
